# The HIV-1 Accessory Protein Vpu Downregulates Peroxisome Biogenesis

**DOI:** 10.1128/mBio.03395-19

**Published:** 2020-03-03

**Authors:** Zaikun Xu, Robert Lodge, Christopher Power, Eric A. Cohen, Tom C. Hobman

**Affiliations:** a Department of Cell Biology, University of Alberta, Edmonton, Alberta, Canada; b Laboratory of Human Retrovirology, Institut de Recherches Cliniques de Montréal, Montréal, Quebec, Canada; c Department of Medicine, University of Alberta, Edmonton, Alberta, Canada; d Department of Medical Microbiology and Immunology, University of Alberta, Edmonton, Alberta, Canada; e Li Ka Shing Institute of Virology, University of Alberta, Edmonton, Alberta, Canada; f Women & Children's Health Research Institute, University of Alberta, Edmonton, Alberta, Canada; g Neuroscience and Mental Health Institute, University of Alberta, Edmonton, Alberta, Canada; h Department of Microbiology, Infectious Diseases and Immunology, Université de Montréal, Montréal, Quebec, Canada; Johns Hopkins Medical School; Johns Hopkins Bloomberg School of Public Health

**Keywords:** HIV, Vpu, peroxisomes, miRNAs, β-catenin

## Abstract

Human immunodeficiency virus type 1 (HIV-1) establishes lifelong infections in humans, a process that relies on its ability to thwart innate and adaptive immune defenses of the host. Recently, we reported that HIV-1 infection results in a dramatic reduction of the cellular peroxisome pool. Peroxisomes are metabolic organelles that also function as signaling platforms in the innate immune response. Here, we show that the HIV-1 accessory protein Vpu is necessary and sufficient for the depletion of cellular peroxisomes during infection. Vpu induces the expression of four microRNAs that target mRNAs encoding proteins required for peroxisome formation and metabolic function. The ability of Vpu to downregulate peroxisomes was found to be dependent upon the Wnt/β-catenin signaling pathway. Given the importance of peroxisomes in innate immune signaling and central nervous system function, the roles of Vpu in dampening antiviral signaling appear to be more diverse than previously realized. Finally, our findings highlight a potential role for Wnt/β-catenin signaling in peroxisome homeostasis through modulating the production of biogenesis factors.

## Abstract (precis)

People living with HIV can experience accelerated aging and the development of neurological disorders. Recently, we reported that HIV-1 infection results in a dramatic loss of peroxisomes in macrophages and brain tissue. This is significant because (i) peroxisomes are important for the innate immune response and (ii) loss of peroxisome function is associated with cellular aging and neurodegeneration. Accordingly, understanding how HIV-1 infection causes peroxisome depletion may provide clues regarding how the virus establishes persistent infections and, potentially, the development of neurological disorders. Here, we show that the accessory protein Vpu is necessary and sufficient for the induction of microRNAs that target peroxisome biogenesis factors. The ability of Vpu to downregulate peroxisome formation depends on the Wnt/β-catenin pathway. Thus, in addition to revealing a novel mechanism by which HIV-1 uses intracellular signaling pathways to target antiviral signaling platforms (peroxisomes), we have uncovered a previously unknown link between the Wnt/β-catenin pathway and peroxisome homeostasis.

## INTRODUCTION

Viruses have evolved intricate strategies to interfere with and/or activate host cell pathways in order to facilitate replication and release of new virions. Human immunodeficiency virus type 1 (HIV-1) causes chronic infections in its human hosts, a situation that necessitates highly effective and often redundant mechanisms to thwart antiviral signaling. In addition to compromised immune system function, a significant fraction of HIV-infected patients (∼25%) develop a spectrum of cognitive, motor, and/or behavioral impairments (1, 2). The development of these neurological deficits is linked to viral and host genetic factors as well as immune factors (3–8) and side effects of antiretroviral therapies (9). Host genetic factors whose altered expression is associated with HIV-induced neurocognitive defects include microRNAs (miRNAs) (10).

We recently reported that HIV-1 infection upregulates several miRNAs that affect peroxisome formation (11). Specifically, increased expression of miR-500a-5p, miR-34c-3p, miR-93-3p, and miR-381-3p was observed in the brains of HIV patients with neurocognitive deficits. These miRNAs downregulate the expression of PEX2, -7, -11B, and -13, which are proteins required for the biogenesis of peroxisomes (reviewed in reference 12). Within the peroxisomal matrix, more than 50 different enzymes perform critical metabolic functions, including the production and degradation of hydrogen peroxide, oxidation of fatty acids, and synthesis of specialized lipids, all of which are important for the development and function of the central nervous system (13). Over the last decade, peroxisomes have emerged as signaling hubs for antiviral defense pathways, including the interferon response (14, 15). Moreover, mounting evidence indicates that viruses employ a variety of strategies to interfere with peroxisome-dependent antiviral signaling. For example, flaviviruses such as West Nile and dengue viruses eliminate peroxisomes in part by capsid protein-dependent sequestration and degradation of the peroxisomal biogenesis factor PEX19 (16). Hepatitis C virus, a distantly related *Flaviviridae* member, reduces antiviral signaling by targeting the pool of mitochondrial antiviral signaling protein on peroxisomes for cleavage by the NS3-4A protease (17–19). Whether peroxisomes are depleted is not known, but a recent study showed that the β-oxidation function of these organelles is impaired during hepatitis C virus infection (20). Downregulation of peroxisomes was also observed during HIV-1 infection of monocyte-derived macrophages (MDMs) (11), suggesting that targeting these organelles is a key aspect of HIV biology.

In the present study, we investigated how HIV-1 infection leads to increased expression of PEX mRNA-targeting miRNAs and, ultimately, the loss of peroxisomes. Our data indicate that the viral accessory protein Vpu is both necessary and sufficient for this process. Vpu has long been known to modulate the expression of specific host plasma membrane proteins and functions in virion release (reviewed in reference 21). More recently, though, this viral protein was shown to suppress antiviral genes, including interferon beta, by inhibiting the transcription factor NF-κB (22). Given the roles of peroxisomes in innate immune signaling and central nervous system function, the manner in which Vpu dampens antiviral signaling appears to be even broader in scope than previously realized. Finally, as peroxisomes play critical roles in brain function (23) and modulating lipotoxicity (24–26), it is tempting to speculate that Vpu-dependent loss of peroxisome function plays a role in virus-associated neuropathogenesis as well as metabolic syndrome and/or lipodystrophy.

## RESULTS

### Vpu is required for downregulation of peroxisomes.

**FIG 1:**
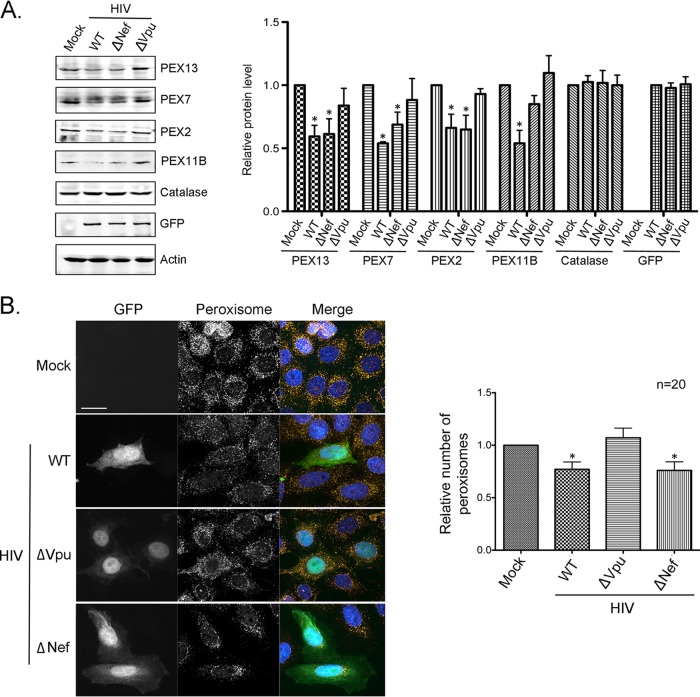
Vpu is required for downregulation of peroxisomes. (A) HeLa-CD4/CXCR4/CCR5 cells were infected with either WT HIV-1 (NL4-3ADA.GFP), Δ*Nef*, or Δ*Vpu* viruses for 72 h, after which cell lysates were subjected to immunoblot analyses with antibodies to PEX2, PEX7, PEX11B, PEX13, catalase, GFP, and actin. The relative levels of peroxisomal proteins (compared to actin) from 3 independent experiments were averaged and plotted. Error bars represent standard errors of the means. (B) HeLa-CD4/CXCR4/CCR5 cells were infected with the above-described viruses (MOI = 2) for 72 h and then processed for indirect immunofluorescence and confocal microscopy. Peroxisomes were detected with a mouse monoclonal antibody to PMP70 and donkey anti-mouse IgG conjugated to Alexa Fluor 546. HIV-infected cells were detected by GFP. Nuclei were stained using DAPI. Images were obtained using a spinning-disc confocal microscope. Bar = 10 μm. The numbers of peroxisomes (PMP70-positive structures) in mock- and HIV-infected cells were determined using Volocity image analysis software. Averages were calculated from three independent experiments in which a minimum of 5 cells for each sample were analyzed. The average number in mock-treated cells was normalized to 1. Bars represent standard errors of the means. *, *P* < 0.05.

HIV-1 infection of brain tissue, MDMs, and immortalized human cell lines results in a significant loss of peroxisomal proteins (11). While it was determined that HIV-induced upregulation of host miRNAs was required for this process, the viral protein(s) responsible for peroxisome loss had not been identified. As a first step in determining which HIV protein(s) was responsible for this phenomenon, HeLa-CD4/CXCR4/CCR5 cells (27) were infected with green fluorescent protein (GFP)-tagged HIV-1 lacking coding regions for the accessory protein Nef or Vpu (28). Seventy-two hours later, the relative levels of four peroxisomal biogenesis factors (PEX2, PEX7, PEX11B, and PEX13) that are downregulated during HIV-1 infection of human cells were assessed by immunoblotting. Data in Fig. 1A show that infection with wild-type (WT) HIV-1 resulted in 30 to 45% reductions in PEX2, PEX7, PEX11B, and PEX13 protein levels. The fact that infection did not affect the levels of the peroxisomal matrix enzyme catalase indicates that the effect of the virus on peroxisomal proteins is highly specific. Infection with an isogenic *Nef* knockout virus had a similar effect on PEX2, PEX7, PEX13, and PEX11B as wild-type HIV-1. In contrast, infection with an isogenic *Vpu* knockout virus did not result in significantly reduced levels of these proteins compared to mock-infected samples. The lack of an effect of the *Vpu* knockout virus on peroxisome biogenesis factors was not due to reduced replication, as levels of GFP expression were similar among HeLa-CD4/CXCR4/CCR5 cells infected with the three different viruses (Fig. 1A).

In parallel, the numbers of peroxisomes in HeLa-CD4/CXCR4/CCR5 cells infected with GFP-tagged HIV lacking *Nef* or *Vpu* were quantified. Data in Fig. 1B show representative confocal images of peroxisomes in cells at 72 h postinfection. Quantitation of the PMP70-positive puncta revealed that infection with the *Vpu* knockout virus did not deplete peroxisomes, whereas wild-type and *Nef* knockout viruses reduced the peroxisome pool by ∼25% (Fig. 1B).

To assess whether Vpu was required for the loss of peroxisomal proteins in primary human cells, relative levels of PEX2, PEX7, PEX11B, and PEX13 were determined in HIV-1-infected monocyte-derived macrophages. Immunoblot data in Fig. S1A in the supplemental material show that infection of macrophages with the *Vpu* deletion virus did not deplete peroxisome biogenesis factors. Conversely, infection of these cells with wild-type HIV-1 or an isogenic *Nef* knockout virus resulted in 40 to 50% reductions in the levels of peroxisome biogenesis factors.

10.1128/mBio.03395-19.1 FIG S1 Vpu is required for the downregulation of peroxisomal proteins and the induction of miRNAs that target mRNAs encoding peroxisome biogenesis factors in infected macrophages. (A) Monocyte-derived macrophages (MDMs) were infected with wild-type (WT) HIV-1 (NL4-3ADA.GFP) or HIV-1 defective in Nef or Vpu expression (Δ*Nef* or Δ*Vpu*) for 72 h, after which cell lysates were subjected to immunoblot analyses with antibodies to PEX2, PEX7, PEX11B, PEX13, GFP, and actin. The relative levels of peroxisomal proteins (compared to actin) from 3 independent experiments were averaged and are plotted. Error bars represent standard errors of the means. (B) MDMs were infected with the above-described viruses for 72 h. The relative levels of miRNAs were then determined by RT-qPCR from total RNA extracted from the samples. The average relative levels of miRNAs (normalized to snRNU6) from 3 independent experiments were determined. Error bars represent standard errors of the means. *, *P* < 0.05. Download FIG S1, TIF file, 0.5 MB. Copyright © 2020 Xu et al. 2020 Xu et al. This content is distributed under the terms of the Creative Commons Attribution 4.0 International license.

The upregulation of miRNAs that target mRNAs encoding peroxisome biogenesis factors is associated with peroxisome loss during HIV-1 infection (11). Accordingly, we monitored the levels of miR-500a-5p, miR-34c-3p, miR-93-3p, and miR-381-3p in macrophages infected with wild-type, Δ*Vpu*, and Δ*Nef* viruses of HIV-1. Total RNA from mock-treated and infected macrophages was extracted at 72 h postinfection, and relative levels of miRNAs were determined by reverse transcription-quantitative PCR (RT-qPCR). Data in Fig. S1B show that the expression of PEX mRNA-targeting miRNAs (miR-500a-5p, miR-34c-3p, miR-93-3p, and miR-381-3p) was elevated 2.2- to 3.1-fold in macrophages infected with wild-type or Δ*Nef* HIV-1. In contrast, the levels of these miRNAs were not significantly affected by infection with the Δ*Vpu* virus, thus establishing a link between the presence of Vpu and the upregulation of PEX mRNA-targeting miRNAs. Together, these data indicate that Vpu is necessary for the HIV-induced loss of peroxisomes.

### Vpu expression is sufficient for downregulation of peroxisomes.

**FIG 2:**
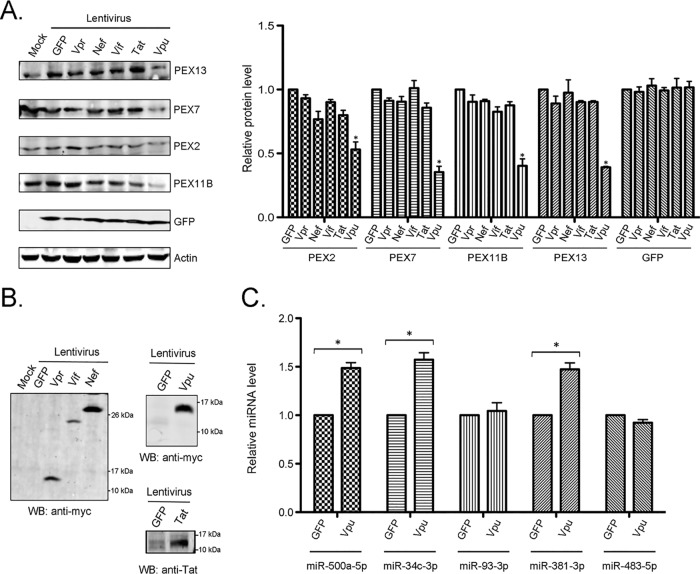
Vpu expression induces the expression of miRNAs that downregulate peroxisome biogenesis factors. (A) HeLa-CD4/CXCR4/CCR5 cells were transduced with GFP-expressing lentiviruses encoding HIV accessory or regulatory proteins (either Vpr, Vpu, Nef, Vif, or Tat) for 48 h, after which cell lysates were subjected to immunoblot analyses with antibodies to PEX2, PEX7, PEX11B, PEX13, GFP, and actin. The relative levels of specific peroxisomal proteins (compared to actin) from 3 independent experiments were averaged and plotted. Error bars represent standard errors of the means. (B) The expression of viral proteins was confirmed by immunoblotting with antibodies against myc or Tat. WB, Western blot. (C) HeLa-CD4/CXCR4/CCR5 cells were transduced with lentiviruses expressing GFP, or GFP and Vpu, for 48 h. Relative levels of miRNAs were determined by RT-qPCR from total RNA extracted from the samples. The average relative levels of miRNAs (normalized to snRNU6) from 3 independent experiments were determined. Error bars represent standard errors of the means. *, *P* < 0.05.

To determine how the expression of Vpu in the absence of other viral proteins affected peroxisomal proteins, HeLa-CD4/CXCR4/CCR5 cells were transduced with GFP-expressing lentiviruses encoding the myc-tagged HIV-1 accessory proteins Vpu, Vpr, Nef, and Vif or the regulatory protein Tat. Data in Fig. 2A show that based on GFP expression, similar levels of transduction were achieved with each of the lentiviruses. Expression of the HIV accessory proteins Vpr, Vif, Nef, and Vpu was confirmed by immunoblotting with an antibody to the myc epitope, while Tat expression was verified by using an anti-Tat antibody (Fig. 2B). Whereas Vpu expression reduced the steady-state levels of PEX2, PEX7, PEX11B, and PEX13, neither Vpr, Nef, Vif, nor Tat significantly affected the levels of these proteins compared to those in negative-control cells expressing GFP only (Fig. 2A).

Next, we investigated whether the expression of Vpu increased the levels of miRNAs that suppress the expression of PEX2, PEX7, PEX11B, and PEX13 (11). Relative levels of miR-500a-5p, miR-34c-3p, miR-93-3p, and miR-381-3p were determined by RT-qPCR at 48 h posttransduction. Data in Fig. 2C show that with the exception of miR-93-3p, levels of PEX-targeting miRNAs (miR-500a-5p, miR-34c-3p, and miR-381-3p) were elevated between 1.5- and 1.7-fold in Vpu-expressing cells. This is consistent with our previous observation that HIV-1 infection of HeLa-CD4/CXCR4/CCR5 cells upregulates the expression of miR-500a-5p, miR-34c-3p, and miR-381-3p but not miR-93-3p (11). Conversely, infection of macrophages with HIV-1 was associated with increased levels of miR-93-3p (Fig. S1B). Levels of the control, miR-483-5p, which does not target PEX mRNAs, were not affected by Vpu expression (Fig. 2C).

We were at first puzzled by the fact that Vpu expression reduces the steady-state level of PEX11B in HeLa-CD4/CXCR4/CCR5 cells even though the miRNA (miR-93-3p) that targets PEX11B mRNA was not upregulated in these cells. This is likely due to the fact that steady-state levels of PEX proteins can be negatively affected by the loss of one or more of the other PEX proteins (29–31). Moreover, we previously showed that small interfering RNAs (siRNAs) against PEX7 mRNA also reduced the levels of PEX11B protein (11). As such, the loss of PEX11B in HIV-1-infected macrophages may be due to increased expression of miR-93-3p (which targets PEX11B mRNA) and miR-34c-3p (which targets PEX7 mRNA).

**FIG 3:**
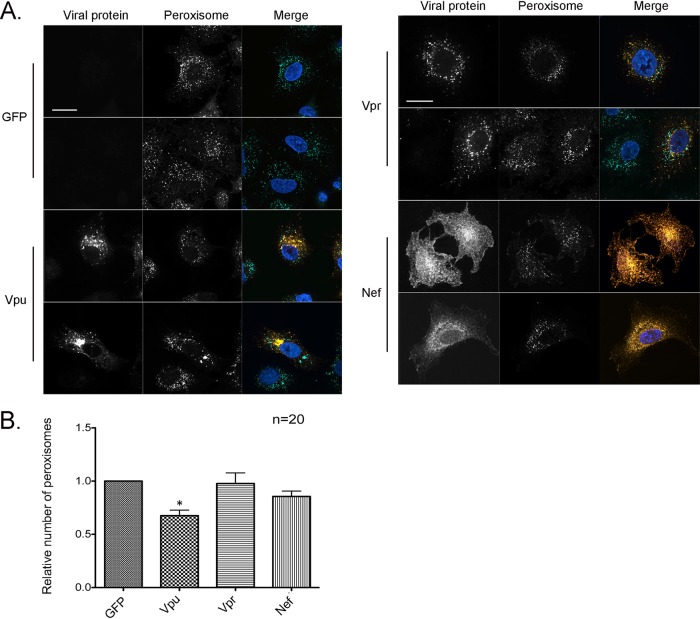
Vpu expression results in the depletion of peroxisomes. (A) HeLa-CD4/CXCR4/CCR5 cells were transduced with lentiviruses expressing either Vpu, Vpr, or Nef for 48 h and then processed for indirect immunofluorescence and confocal microscopy. Peroxisomes were detected with a rabbit polyclonal antibody to the peroxisome-targeting signal SKL and donkey anti-rabbit IgG conjugated to Alexa Fluor 488. Viral protein-transduced cells were detected with a mouse monoclonal antibody to the myc tag and donkey anti-mouse IgG conjugated to Alexa Fluor 546. Nuclei were stained using DAPI. Images were obtained using a spinning-disc confocal microscope. Bar = 10 μm. (B) The numbers of peroxisomes (SKL-positive structures) in GFP-only- and HIV protein-transduced cells were determined using Volocity image analysis software. Averages were calculated from three independent experiments in which a minimum of 5 cells for each sample were analyzed. The average number in GFP-treated cells was normalized to 1. Bars represent standard errors of the means. *, *P* < 0.05.

Finally, the numbers of peroxisomes were quantified in HeLa-CD4/CXCR4/CCR5 cells 48 h after transduction with lentiviruses encoding Vpu or other HIV accessory proteins. Data in Fig. 3A show representative confocal images of peroxisomes in these cells. Quantitation of the tripeptide SKL (serine-lysine-leucine)-positive puncta revealed that Vpu but not Vpr or Nef expression reduced the peroxisome abundance by >30% relative to that in cells transduced with a lentivirus encoding GFP alone (Fig. 3B). Together, these data indicate that the expression of Vpu is sufficient for the HIV-induced loss of peroxisomes.

### Vpu mutants that cannot bind β-TrCP do not downregulate peroxisomes.

It is well documented that Vpu promotes the release of virus particles and modulates multiple host cell plasma membrane proteins, including CD4 and BST2 (reviewed in reference 21). More recent evidence indicates that this HIV-1 protein also dampens antiviral signaling by stabilizing IκBα, a negative regulator of NF-κB (22). Recruitment of the β-transducin repeat-containing protein (β-TrCP) component of the Skp, Cullin, F-box (SCF^β-TrCP^) E3 ubiquitin ligase by Vpu leads to proteasome-dependent degradation of newly synthesized CD4 and turnover of BST2 in lysosomes, processes that require casein kinase II-mediated phosphorylation of two serine residues (S52/S56) in Vpu (32–36). Sequestration of β-TrCP by Vpu stabilizes other β-TrCP substrates, including IκBα, ATF4, and β-catenin, that affect cellular transcription (37–39).

**FIG 4:**
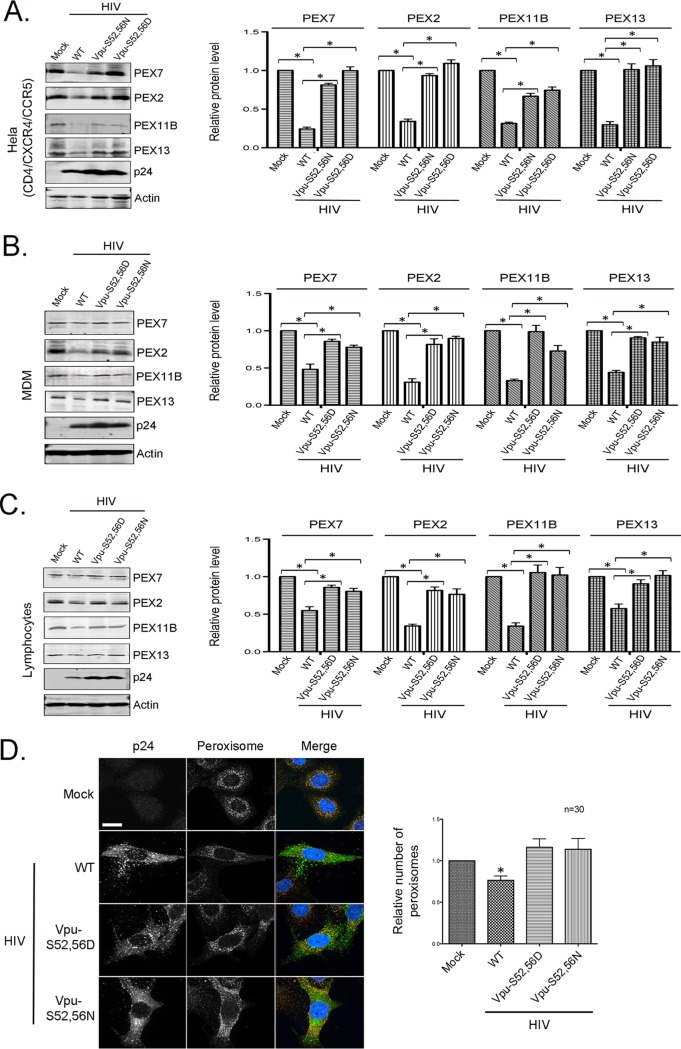
HIV-1 encoding Vpu mutants that cannot sequester β-TrCP does not downregulate peroxisomes. (A to C) HeLa-CD4/CXCR4/CCR5 cells (A), MDMs (B), or lymphocytes (C) were infected with HIV (NL4-3) expressing WT Vpu or Vpu phosphorylation mutants (S52,56D and S52,56N), which do not bind β-TrCP, for 72 h (MOI = 2), after which cell lysates were subjected to immunoblot analyses with antibodies to PEX2, PEX7, PEX11B, PEX13, HIV p24, and actin. The relative levels of peroxisomal proteins (compared to actin) from 3 independent experiments were averaged and plotted. Error bars represent standard errors of the means. (D) HeLa-CD4/CXCR4/CCR5 cells were infected with the above-described viruses (MOI = 2) and then processed for indirect immunofluorescence and confocal microscopy. Peroxisomes were detected with a rabbit polyclonal antibody to the peroxisome-targeting signal SKL and donkey anti-rabbit IgG conjugated to Alexa Fluor 546. HIV-infected cells were detected with a mouse monoclonal antibody to p24 and donkey anti-mouse IgG conjugated to Alexa Fluor 488. Nuclei were stained using DAPI. Images were obtained using a spinning-disc confocal microscope. Bar = 10 μm. The numbers of peroxisomes (SKL-positive structures) in mock- and HIV-infected cells were determined using Volocity image analysis software. Averages were calculated from three independent experiments in which a minimum of 10 cells for each sample were analyzed. The average number in mock-treated cells was normalized to 1. Bars represent standard errors of the means. *, *P* < 0.05.

To determine if peroxisomes were affected by HIV-1 harboring mutated *Vpu* (S52,56D and S52,56N), immunoblot and immunofluorescence assays were conducted on infected HeLa-CD4/CXCR4/CCR5 cells and primary human cells (lymphocytes and MDMs). Unlike wild-type HIV-1, infection with Vpu-S52,56D and Vpu-S52,56N viruses did not result in a significant loss of PEX2, PEX7, PEX11B, or PEX13 in HeLa-CD4/CXCR4/CCR5 cells, macrophages, or T cells (Fig. 4). This was not due to decreased viral infection because in all cases, levels of p24 protein were in fact slightly higher in cells infected with Vpu mutant viruses.

Next, the numbers of peroxisomes were quantitated in HeLa-CD4/CXCR4/CCR5 cells infected with wild-type, Vpu-S52,56D, and Vpu-S52,56N viruses. Similar to what we observed with the Δ*Vpu* virus (Fig. 1), infection with Vpu-S52,56D and Vpu-S52,56N viruses did not result in a loss of peroxisomes (Fig. 4D). Finally, levels of PEX mRNA-targeting miRNAs in cells infected with wild-type, Vpu-S52,56D, and Vpu-S52,56N viruses were determined by RT-qPCR. Consistent with the data showing a lack of an effect on peroxisome biogenesis factor levels, infection with HIV-1 harboring mutated Vpu did not significantly increase the expression of miR-500a-5p, miR-34c-3p, miR-93-3p, or miR-381-3p in HeLa-CD4/CXCR4/CCR5 cells, macrophages, or T cells (Fig. S2). Interestingly, modest but statistically significant decreases in the levels of miR-483-5p (which does not target PEX mRNAs) were observed in some of the infected samples (Fig. S1B and Fig. S2A to C). However, in no cases did we observe a significant upregulation of this miRNA in any infected or Vpu-transfected samples.

10.1128/mBio.03395-19.2 FIG S2 HIV-1 encoding Vpu phosphomutants does not increase the expression of miRNAs that suppress peroxisome biogenesis factors. HeLa-CD4/CXCR4/CCR5 cells (A), MDMs (B), or lymphocytes (C) were infected with HIV (NL4-3) encoding wild-type Vpu or the indicated mutant Vpu for 72 h (MOI = 2). Relative levels of miRNAs were determined by RT-qPCR from total RNA extracted from the samples. The average relative levels of miRNAs (normalized to snRNU6) from 3 independent experiments were determined. Error bars represent standard errors of the means. *, *P* < 0.05. Download FIG S2, TIF file, 0.4 MB. Copyright © 2020 Xu et al. 2020 Xu et al. This content is distributed under the terms of the Creative Commons Attribution 4.0 International license.

Data from the infection experiments were further corroborated by the expression of Vpu-S52,56D (40) in HeLa-CD4/CXCR4/CCR5 cells. Specifically, the expression of this Vpu mutant did not reduce the levels of PEX2, PEX7, PEX11B, or PEX13 proteins (Fig. S3A) or induce the expression of PEX-targeting miRNAs (Fig. S3B). Similarly, the cellular peroxisome pool was not significantly reduced by the expression of Vpu-S52,56D (Fig. S3C).

10.1128/mBio.03395-19.3 FIG S3 A Vpu mutant that cannot sequester β-TrCP does not downregulate peroxisomes. (A) HeLa-CD4/CXCR4/CCR5 cells were transfected with AU1-tagged WT Vpu or Vpu-S52,56D (which does not bind β-TrCP) for 48 h, after which cell lysates were subjected to immunoblot analyses with antibodies to PEX2, PEX7, PEX11B, PEX13, AU1, and actin. The relative levels of peroxisomal proteins (compared to actin) from 3 independent experiments were averaged and are plotted. Error bars represent standard errors of the means. *, *P* < 0.05. (B) HeLa-CD4/CXCR4/CCR5 cells were transfected with wild-type or S52,56D mutant Vpu for 48 h. Relative levels of miRNAs were determined by qPCR from total RNA extracted from the samples. The average relative levels of miRNAs (normalized to snRNU6) from 3 independent experiments were determined. Error bars represent standard errors of the means. *, *P* < 0.05. (C) HeLa-CD4/CXCR4/CCR5 cells were transfected with the above-described constructs for 48 h and then processed for indirect immunofluorescence and confocal microscopy. Peroxisomes were detected with a mouse monoclonal antibody to PMP70 and donkey anti-mouse IgG conjugated to Alexa Fluor 488. Vpu-transfected cells were detected by using rabbit polyclonal antibody to AU1 and donkey anti-rabbit IgG conjugated to Alexa Fluor 546. Nuclei were stained using DAPI. Images were obtained using a spinning-disc confocal microscope. Bar = 10 μm. The numbers of peroxisomes (PMP70-positive structures) in WT or mutant Vpu-transfected cells were determined using Volocity image analysis software. Averages were calculated from three independent experiments in which a minimum of 8 cells for each sample were analyzed. The average number in mock-transfected cells was normalized to 1. Bars represent standard errors of the means. *, *P* < 0.05. Download FIG S3, TIF file, 1.5 MB. Copyright © 2020 Xu et al. 2020 Xu et al. This content is distributed under the terms of the Creative Commons Attribution 4.0 International license.

**FIG 5:**
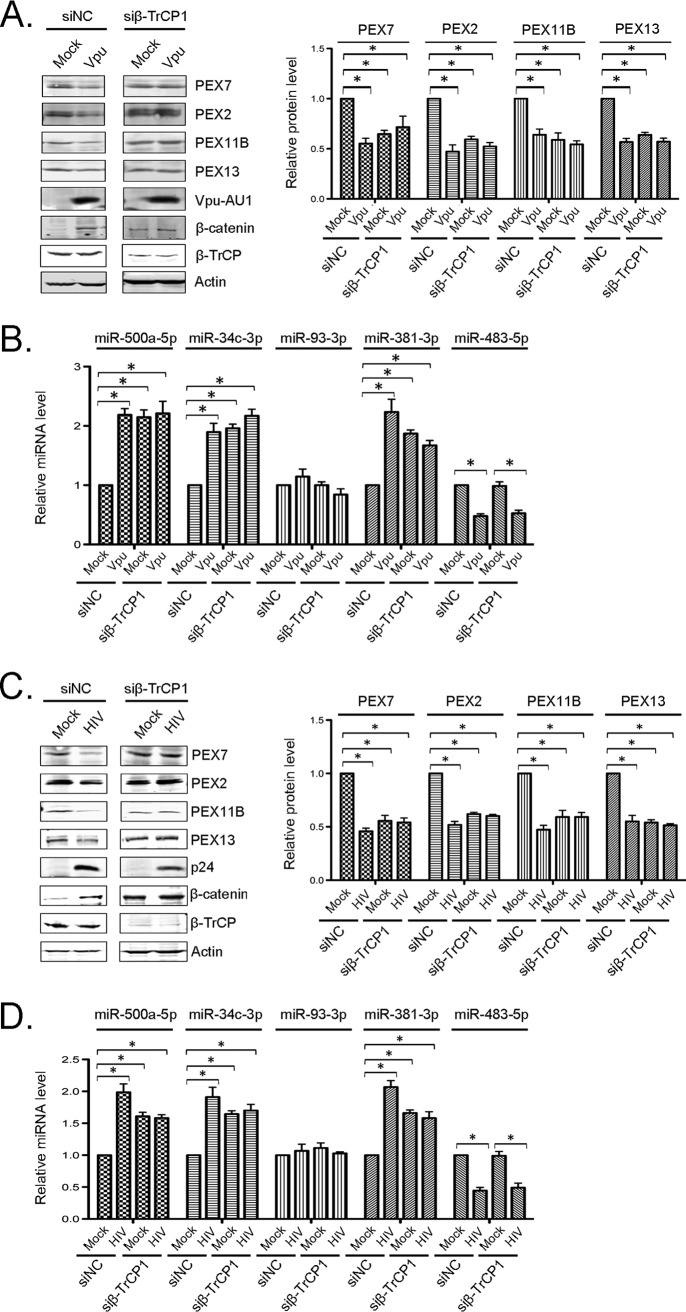
β-TrCP knockdown has a similar effect on peroxisome biogenesis factors as Vpu expression and HIV-1 infection. HeLa-CD4/CXCR4/CCR5 cells were transfected with siRNAs against β-TrCP or a nonsilencing control siRNA (siNC) for 24 h and then retransfected with AU1-tagged WT Vpu for another 48 h (A and B) or infected with HIV-1 (NL4-3) (MOI = 3) for 72 h (C and D). (A and C) Cell lysates were subjected to immunoblot analyses with antibodies to PEX2, PEX7, PEX11B, PEX13, β-TrCP, β-catenin, p24, AU1, and actin. The relative levels of peroxisomal proteins (compared to actin) from 3 independent experiments were averaged and plotted. Error bars represent standard errors of the means. (B and D) Total RNAs, including small RNAs, were extracted from the samples, and relative levels of miRNAs were determined by RT-qPCR. The average relative levels of miRNAs (normalized to snRNU6) from 3 independent experiments were determined. Error bars represent standard errors of the means. *, *P* < 0.05.

Our results are consistent with a scenario in which sequestration of β-TrCP by Vpu is important for downregulating peroxisomes during HIV-1 infection. If so, knockdown of β-TrCP should result in increased expression of miRNAs that suppress peroxisome biogenesis factors independently of Vpu expression or HIV-1 infection. Indeed, lower levels of PEX2, PEX7, PEX11B, and PEX13 proteins were observed in β-TrCP knockdown cells regardless of whether they were transfected with a Vpu expression construct (Fig. 5A) or infected with HIV-1 (Fig. 5C). We also observed elevated expression levels of PEX mRNA-targeting miRNAs (except for miR-93-3p, which is not induced by HIV-1 in HeLa-CD4/CXCR4/CCR5 cells) in β-TrCP knockdown cells (Fig. 5B and D).

### β-Catenin and TCF-4 are required for Vpu-induced downregulation of peroxisomes.

**FIG 6:**
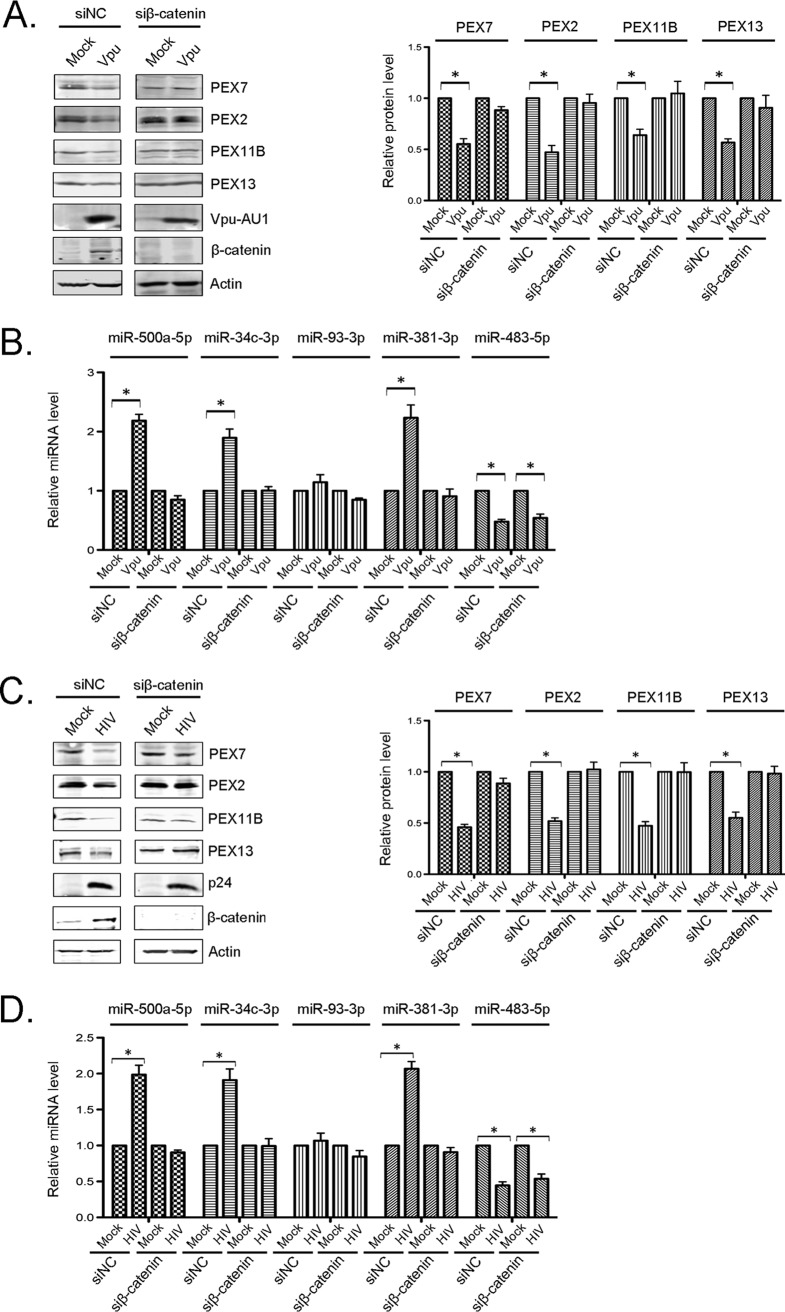
β-Catenin is required for Vpu-induced downregulation of peroxisomes. HeLa-CD4/CXCR4/CCR5 cells were transfected with pooled siRNAs against β-catenin or nonsilencing control siRNAs (siNC) for 24 h and then retransfected with AU1-tagged WT Vpu for another 48 h (A and B) or infected with HIV-1 (NL4-3) (MOI = 3) for 72 h (C and D). (A and C) Cell lysates were subjected to immunoblot analyses with antibodies to PEX2, PEX7, PEX11B, PEX13, β-catenin, p24, AU1, and actin. The relative levels of peroxisomal proteins (compared to actin) from 3 independent experiments were averaged and plotted. Error bars represent standard errors of the means. (B and D) Total RNAs, including small RNAs, were extracted from the samples, and relative levels of miRNAs were determined by RT-qPCR. The average relative levels of miRNAs (normalized to snRNU6) from 3 independent experiments were determined. Error bars represent standard errors of the means. *, *P* < 0.05.

Among the β-TrCP substrates stabilized by Vpu is β-catenin, which is involved in cellular transcription (37–39). When stabilized, β-catenin translocates from the cytoplasm to the nucleus, where it associates with members of the T-cell factor (TCF) DNA-binding proteins to activate the transcription of target genes (reviewed in reference 41). We predicted that β-catenin is necessary for the Vpu-induced loss of peroxisome biogenesis factors, and data in Fig. 6 are consistent with this scenario. Specifically, the expression of Vpu in β-catenin knockdown cells did not reduce the levels of PEX2, PEX7, PEX11B, or PEX13 proteins (Fig. 6A) or induce the expression of PEX mRNA-targeting miRNAs (Fig. 6B). Similarly, infection of β-catenin knockdown cells with wild-type HIV-1 did not affect peroxisome biogenesis factors or PEX mRNA-targeting miRNAs (Fig. 6C and D).

**FIG 7:**
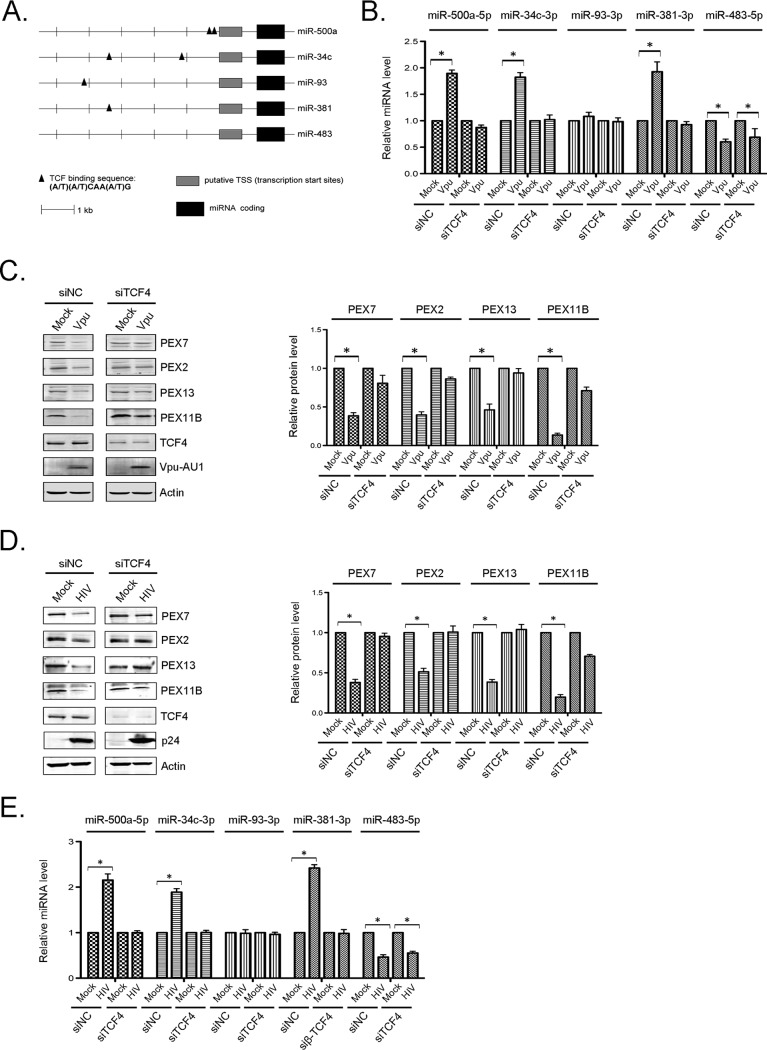
TCF-4 is important for Vpu-mediated downregulation of peroxisome biogenesis factors. (A) Positions of predicted TCF-binding sites upstream of transcriptional start sites in 4 miRNA genes that regulate the translation of peroxisome biogenesis factors. miRNA-483 (which does not target peroxisome biogenesis factors) does not contain TCF-binding sites within its 5′ regulatory region. (B to E) HeLa-CD4/CXCR4/CCR5 cells were transfected with siRNAs against TCF-4 or a nonsilencing control siRNA (siNC) for 24 h and then retransfected with AU1-tagged WT Vpu for another 48 h (B and C) or infected with HIV-1 (NL4-3) (MOI = 3) for 72 h (D and E). (B and E) Total RNAs, including small RNAs, were extracted from the samples, and relative levels of miRNAs were determined by RT-qPCR. The average relative levels of miRNAs (normalized to snRNU6) from 3 independent experiments were determined. Error bars represent standard errors of the means. (C and D) Cell lysates were subjected to immunoblot analyses with antibodies to PEX2, PEX7, PEX11B, PEX13, TCF-4, p24, AU1, and actin. The relative levels of peroxisomal proteins (compared to actin) from 3 independent experiments were averaged and plotted. Error bars represent standard errors of the means. *, *P* < 0.05.

β-Catenin/TCF complexes bind to the consensus sequence (A/T)(A/T)CAA(A/T)G in the upstream regions of target genes (42). Analyses of the 5′ regulatory regions of miR-500a-5p, miR-34c-3p, miR-93-3p, and miR-381-3p revealed that they all contain at least one TCF-binding site (Fig. 7A). Conversely, TCF-binding sites were not present upstream of the transcriptional start site of miR-483-5p, a microRNA that is not upregulated by Vpu. TCF-4 is considered the most important transcription factor in the TCF/LEF (lymphoid enhancer-binding factor) family, as it cooperates with β-catenin to activate the transcription of many genes, including those that drive tumorigenesis (reviewed in reference 43). Similar to data in Fig. 6 showing that β-catenin is required for Vpu-mediated depletion of peroxisomal proteins, the expression of Vpu (Fig. 7B and C) or HIV-1 infection (Fig. 7D and E) of TCF-4 knockdown cells did not result in the upregulation of miRNAs that downregulate peroxisome biogenesis factors. Together, these data indicate that the Wnt/β-catenin pathway is required for Vpu-mediated depletion of peroxisomes during HIV-1 infection.

### Vpu expression leads to increased levels of nonesterified fatty acids.

**FIG 8:**
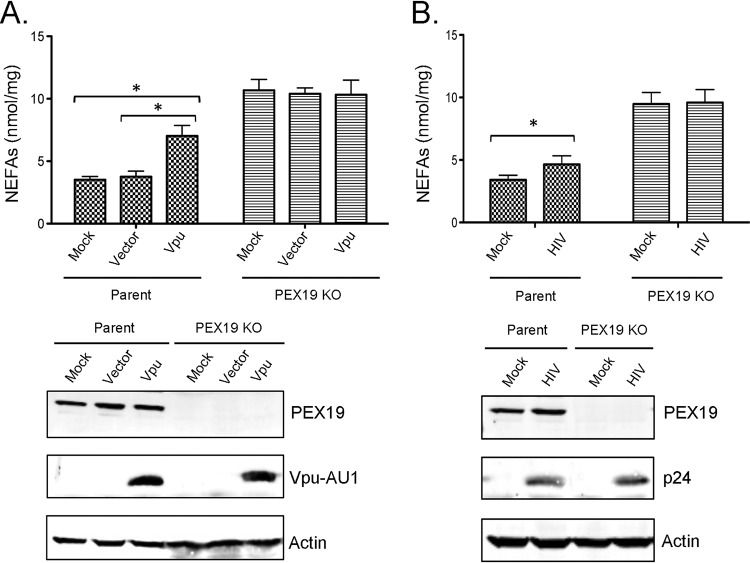
Vpu- and HIV-induced downregulation of peroxisomes and accumulation of NEFAs. (A) Parent and PEX19 knockout (KO) HeLa-CD4/CXCR4/CCR5 cells were transfected with pCGCG-Vpu-AU1 or an empty vector (pCGCG) for 48 h. At 48 h posttransfection, the levels of NEFAs relative to the protein content in each sample were determined. (B) Parent and PEX19 knockout HeLa-CD4/CXCR4/CCR5 cells were infected with HIV-1 (NL4-3) (MOI = 3) for 72 h, after which the levels of NEFAs relative to the protein content in each sample were determined. Values reported represent the averages of data from three independent experiments. Error bars represent standard errors of the means. *, *P* < 0.05.

Peroxisomes are critical for modulating the levels of nonesterified fatty acids (NEFAs) in eukaryotic cells, a metabolic function that is important for protection against lipotoxicity and oxidative stress (24–26). To assess the potential effect of Vpu expression on peroxisome function, the concentrations of NEFAs in Vpu-transfected and HIV-1-infected HeLa-CD4/CXCR4/CCR5 cells were determined. Data in Fig. 8A show that compared to mock- or vector-transfected cells, the levels of NEFAs in Vpu-expressing cells were increased 2-fold. As a positive control for these experiments, we used CRISPR to create a strain of CD4/CXCR4/CCR5 cells devoid of peroxisomes due to a lack of PEX19. Indeed, levels of NEFAs were 3-fold higher in these cells than in the parent cell line (Fig. 8A). NEFA concentrations were also higher in HIV-1-infected cells (Fig. 8B) but not to the same extent as with Vpu transfectants. This may be because the transfection efficiencies (35 to 40%) were higher than HIV-1 infection rates (10 to 15%) in CD4/CXCR4/CCR5 cells (data not shown). Finally, the fact that neither Vpu expression nor HIV-1 infection affected the levels of NEFAs in PEX19 knockout cells suggests that the Vpu-dependent increase in NEFAs is due to the downregulation of peroxisomes.

## DISCUSSION

HIV-1 Vpu is a multifunctional accessory protein that plays critical roles in virion egress and receptor downregulation (reviewed in reference 21). It also inhibits the expression of antiviral genes by suppressing NF-κB-dependent transcription (22). Dampening the innate immune response is critical for the replication of most if not all viruses that infect mammalian cells. Much of what is known about this process derives from studies of antiviral defense pathways that intersect with mitochondria. These include canonical interferon induction pathways that are activated following the detection of viral RNA by cytosolic RNA helicases such as RIG-I and MDA-5 (44). In addition to mitochondria, peroxisomes are now known to be important hubs for antiviral signaling, and a limited but growing number of studies suggest that the biogenesis of these organelles is inhibited during viral infection (reviewed in reference 45).

We recently reported that HIV-1 interferes with peroxisome functions through a unique mechanism. Rather than directly targeting proteins required for the formation of peroxisomes or antiviral signaling, as is the case for members of the *Flaviviridae* (16, 17), HIV-1 infection induces miRNAs (miR-500a-5p, miR-34c-3p, miR-93-3p, and miR-381-3p) that block the expression of multiple peroxisome biogenesis factors (11). While induction of miR-500a-5p, miR-34c-3p, and miR-381-3p was observed in all cell types examined, increased expression of miR-93-3p was observed only in MDMs and brain tissue infected with HIV-1. In addition to dampening the innate immune response, peroxisome loss during HIV-1 infection may have other consequences. These organelles play critical roles in the development and function of the central nervous system (reviewed in reference 23), and it is worth noting the striking loss of peroxisomal proteins in the brains of HIV patients (11). Moreover, the expression levels of miRNAs that target mRNAs encoding PEX proteins are significantly higher in brain tissue from patients with HIV-associated neurocognitive defects than in HIV patients without dementia (11). As such, elucidating how HIV-1 upregulates the expression of these cellular miRNAs may provide important clues regarding the molecular basis of neuropathological and neuropsychiatric manifestations of HIV infection (“neuro-HIV”). Moreover, in addition to neurological problems, people living with HIV exhibit other morbidities such as metabolic syndrome and lipodystrophy. Both of these conditions are linked to lipotoxicity, a known side effect of some anti-HIV drugs, particularly protease inhibitors (reviewed in references 46 and 47). Peroxisomes also reduce lipotoxicity and oxidative stress in cells by modulating levels of nonesterified fatty acids (24–26), and as such, it is tempting to speculate that the loss of peroxisomes as a result of HIV-1 infection *per se* is a factor contributing to metabolic syndrome and/or lipodystrophy.

Here, we show that Vpu (48, 49) is necessary and sufficient for the induction of cellular miRNAs that target peroxisome biogenesis factor mRNAs. This was demonstrated by infection experiments in primary human macrophages, lymphocytes, and model cell lines as well as by the ectopic expression of Vpu in the absence of other viral proteins. The peroxisome-depleting activity of Vpu is dependent upon the Wnt/β-catenin pathway, which is governed by the phosphorylation status of β-catenin (reviewed in references 50 and 51). Binding of Wnt to the receptors Frizzled and lipoprotein receptor-related protein (LRP) at the cell surface initiates a cascade that allows unphosphorylated cytoplasmic β-catenin to accumulate. After translocation into the nucleus, β-catenin forms complexes with members of the TCF/LEF family and induces the transcription of target genes that regulate tissue development and homeostasis, cell adherence, and inflammation (50, 52). In addition, the expression of miRNAs can also be affected by Wnt/β-catenin signaling (53, 54).

The effect of Vpu on the Wnt/β-catenin pathway requires the sequestration of the β-TrCP subunit of the SCF ubiquitin ligase complex. In fact, the knockdown of β-TrCP had a similar effect on peroxisomes as Vpu expression and HIV-1 infection. Vpu-mediated sequestration of β-TrCP results in elevated levels of β-TrCP substrates, including β-catenin (38). Infection with HIV-1 encoding Vpu mutants that cannot sequester β-TrCP did not induce PEX mRNA-targeting miRNAs, and consequently, peroxisomes were not affected. Similarly, the expression of Vpu in β-catenin knockdown cells did not induce miRNAs that target peroxisome biogenesis factors.

**FIG 9:**
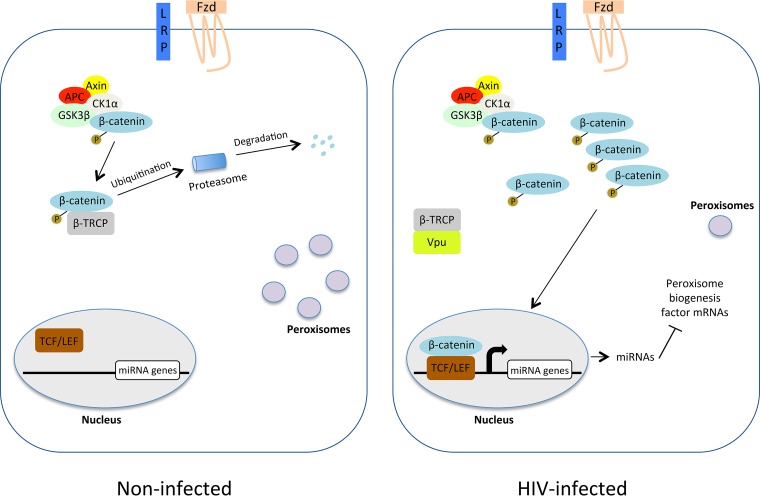
Proposed mechanism by which Vpu downregulates peroxisomes. In the absence of Wnt ligand interaction with Frizzled (Fzd) and lipoprotein receptor-related protein (LRP) at the cell surface, levels of cytoplasmic β-catenin are very low due to a destruction complex that includes adenomatosis polyposis coli (APC), axin, casein kinase 1α (CK1α), and glycogen synthase kinase 3β (GSK3β). β-Catenin is phosphorylated by GSK3β, ubiquitinated in a β-TrCP-dependent manner, and then degraded by the proteasome. During HIV-1 infection, the sequestration of β-TrCP by Vpu results in the stabilization of β-catenin, followed by its translocation into the nucleus. Here, β-catenin forms complexes with TCF/LEF family members that stimulate the transcription of genes encoding miRNAs that inhibit the translation of peroxisome biogenesis factors.

Bioinformatic analyses of the upstream regulatory regions of the miR-500a-5p, miR-34c-3p, miR-93-3p, and miR-381-3p genes revealed potential binding sites for TCF family members, suggesting that the transcription of these miRNAs is modulated by β-catenin and at least one of its binding partners. Indeed, TCF-4, the most important member of this group of transcription factors (43), was found to be important for the Vpu-dependent induction of miRNAs that downregulate peroxisome biogenesis factors. A model depicting how Vpu leads to peroxisome depletion by inducing miRNAs that target peroxisome biogenesis factors is shown in Fig. 9.

While the data presented here are consistent with a scenario in which the Vpu-mediated depletion of peroxisomes would benefit virus replication by dampening the innate immune response, it is worth noting that one group has reported that Wnt/β-catenin signaling restricts HIV-1 in astrocytes (55), blood mononuclear cell preparations (56, 57), and monocytes (58). A limitation of these studies is that lithium chloride was used to activate Wnt/β-catenin signaling. However, as pointed out by those authors, in addition to blocking the activity of glycogen synthase kinase β, which normally downregulates β-catenin, lithium chloride may inhibit HIV-1 replication by affecting other enzymes in mammalian cells (58). Intriguingly, a later study showed that lithium treatment actually results in the hyperphosphorylation of β-catenin in certain cell types (59), a situation that would result in decreased Wnt/β-catenin signaling.

It is important to point out that blood mononuclear cell preparations are generally devoid of macrophages (one of the primary cell types used in the present study). As such, in HIV-permissive target cells (CD4^+^ T cells and macrophages), the effect of Wnt/β-catenin signaling on HIV-1 replication may be different than that in less permissive cell types (astrocytes and monocytes). Indeed, basal β-catenin-mediated transcriptional activity is higher in monocytes than in monocyte-derived macrophages (58), and the possibility that different target genes are activated in these cell types cannot be excluded. Even if Vpu-induced Wnt/β-catenin signaling negatively impacts HIV-1 in some cell types, this may be a trade-off for the potential benefits of counteracting innate immune signaling by depleting peroxisomes. Although it is outside the scope of this study, it will be of interest to determine if specific Vpu genotypes contribute more to the development of neuroHIV by interfering with peroxisome biogenesis.

Finally, our studies may indicate that Wnt/β-catenin signaling is an important modulator of peroxisome homeostasis (in the absence of viral infection). Peroxisome formation occurs through two main pathways, *de novo* biogenesis or growth and division (12). These processes have been extensively characterized over the last 3 decades, and the specific steps in each pathway are well documented. Of note, one of the proteins targeted by HIV-1-induced miRNAs is PEX11B, which functions in the growth and division mode of peroxisome formation. Numerous reports have described the interplay between the Wnt/β-catenin pathway and peroxisome proliferator-activated receptor gamma, a group of transcription factors that regulates glucose metabolism and fatty acid storage (reviewed in reference 60). However, to our knowledge, there is nothing known regarding whether peroxisome proliferator-activated receptor alpha (which stimulates peroxisome formation in mammals) is regulated by the Wnt/β-catenin pathway or vice versa. Because conditions that increase the levels of β-catenin (Vpu expression or knockdown of β-TrCP) in turn lead to the decreased expression of peroxisome biogenesis factors, it is likely that other signals (e.g., Wnt ligands) that activate the Wnt/β-catenin pathway also impair peroxisome biogenesis. Given the critical role of peroxisomes in metabolic processes and neuronal function, pharmacological agents that target Wnt/β-catenin signaling may have benefits for neurological conditions that are associated with the loss of peroxisomes, including HIV-1 infection.

## MATERIALS AND METHODS

### Reagents.

Complete EDTA-free protease inhibitor cocktail was purchased from Roche Diagnostics (Laval, QC, Canada), and ProLong gold antifade reagent with 4′,6-diamidino-2-phenylindole (DAPI), SlowFade gold reagent mounting medium, the cell culture media Dulbecco’s modified Eagle’s medium (DMEM) and RPMI 1640, and fetal bovine serum (FBS) were purchased from Invitrogen (Carlsbad, CA).

Lipofectamine 2000 and Lipofectamine RNAiMAX were purchased from Invitrogen (Carlsbad, CA), and PerFectin transfection reagent was obtained from Genlantis (San Diego, CA). Pooled siRNAs against β-TrCP1 (hs.Ri.BTRC.13) and β-catenin (hs.Ri.CTNNB1.13) or individual siRNAs against TCF-4 (hs.Ri.TCF4.13.1 and hs.Ri.TCF4.13.2) were purchased from IDT (Coralville, IA).

Reagents for the purification and quantitation of miRNAs, including the miRNeasy minikit, the miScript PCR starter kit, the miScript II RT kit, and the miScript SYBR green PCR kit, were purchased from Qiagen (Toronto, ON, Canada).

Chemicals for nonesterified fatty acid (NEFA) extraction, including cupric nitrate trihydrate, triethanolamine, and a mixture of dicarbazone-dicarbazide, were purchased from Sigma (Oakville, Ontario, Canada).

### Antibodies.

Mouse monoclonal antibodies against the peroxisomal membrane protein PMP70 (Sigma, St. Louis, MO), HIV-1 p24 (Abcam, Cambridge, MA), GFP (Abcam), myc (Millipore Sigma, Etobicoke, ON, Canada), and beta-actin (Abcam) were purchased from the indicated suppliers. Rabbit polyclonal antibodies to PEX7, PEX11B, PEX13, PEX19, catalase, beta-catenin, and AU1 were obtained from Abcam; rabbit polyclonal antibody to PEX2 (PXMP3) was purchased from Pierce (Rockford, IL); rabbit monoclonal antibody to β-TrCP (D12C8) was purchased from New England Biolabs (Whitby, ON, Canada); and rabbit polyclonal antibody to the tripeptide SKL was produced as previously described (61).

Donkey anti-mouse IgG conjugated to Alexa Fluor 680, goat anti-rabbit IgG conjugated to Alexa Fluor 800, donkey anti-mouse IgG conjugated to Alexa Fluor 488, donkey anti-rabbit IgG conjugated to Alexa Fluor 488, donkey anti-rabbit IgG conjugated to Alexa Fluor 546, and donkey anti-mouse IgG conjugated to Alexa Fluor 546 were purchased from Invitrogen.

### Isolation and culture of monocyte-derived macrophages and lymphocytes.

Peripheral blood mononuclear cells (PBMCs) from healthy volunteer blood donors (University of Alberta human ethics protocol 00079034) were isolated as previously described (11). Briefly, the blood was diluted 1:1 with phosphate-buffered saline (PBS), placed on top of a layer of Histopaque (Sigma), and centrifuged for 22 min at 665 × *g*. Cells from the interphase layer containing PBMCs were harvested, washed twice with serum-free RPMI 1640, and then resuspended in RPMI 1640 with 15% FBS and 1% penicillin and streptomycin (Invitrogen). The cells (2 million to 4 million per well) were then seeded in 6-well plates that were precoated with poly-l-ornithine (Sigma). After 4 h, media containing lymphocytes were collected and centrifuged. The pelleted lymphocytes were then seeded for infection. Cells that attached to the plates (monocytes) were washed three times with warm RPMI 1640 medium prior to the addition of 2 ml of differentiation medium (25 ng/ml macrophage colony-stimulatory factor [M-CSF] [Sigma] in RPMI 1640 containing 2 mM l-glutamine, 1% penicillin and streptomycin, and 15% FBS) to each well. Cells were incubated for 7 days in this medium (with medium changes every 3 days) to allow differentiation into monocyte-derived macrophages (MDMs).

### Cell culture, transfection, and virus infection.

HEK293T cells from the American Type Culture Collection (Manassas, VA) were cultured in DMEM containing 10% heat-inactivated FBS, 4.5 g/liter d-glucose, 2 mM glutamine, and 110 mg/liter sodium pyruvate at 37°C in a 5% CO_2_ atmosphere. HeLa-CD4/CXCR4/CCR5 cells (27) were cultured in RPMI 1640 supplemented with 10% FBS and 1 mg/ml G418 (Invitrogen).

HeLa-CD4/CXCR4/CCR5 and HEK293T cells were transfected with the expression plasmids using Lipofectamine 2000 (Invitrogen) and PerFectin (Genlantis, San Diego, CA), respectively, as described by the manufacturers. To produce recombinant HIV-1 stocks, HEK293T cells were transfected with proviral constructs (pYU2, pNL4-3, pNL4-3-VpuS52,56D, pNL4-3-VpuS52,56N, pNL4-3ADA.GFP.IRES.Nef, pNL4-3ADA.GFP.IRES.Δ*Nef*, or pNL4.3ADA.GFP.IRES.NefΔ*Vpu*) using PerFectin transfection reagent. At 48 h posttransfection, supernatants containing viruses were cleared of cells and debris, filtered, aliquoted, and kept at −80°C. Virus titers were determined using the HIV-1 p24 antigen capture assay (ABL, Rockville, MD, USA).

Infection of HeLa-CD4/CXCR4/CCR5 cells, primary MDMs, or lymphocytes with HIV-1 was performed under biosafety containment level 3 (CL3) conditions at a multiplicity of infection (MOI) of 2 unless indicated otherwise.

HeLa-CD4/CXCR4/CCR5 cells were transfected with siRNAs (30 nM) against β-TrCP1, β-catenin, or TCF-4 using Lipofectamine RNAiMAX (Invitrogen) as described by the manufacturer.

### Construction of lentiviral plasmids expressing HIV-1 Vpu, Vpr, Vif, Nef, and Tat.

Plasmids were constructed using PCR and standard subcloning techniques. The primers used for the PCRs are listed in Table 1. All constructs were verified by diagnostic restriction endonuclease digestion and DNA sequencing. With the exception of *Tat*, cDNA fragments for myc-tagged viral genes, including *Vpu*, *Vpr*, *Vif*, and *Nef*, were generated by PCR using the HIV proviral DNA NL4-3 (28) as a template with the appropriate primers listed in Table 1. The full-length Tat cDNA was generated by PCR using the SVCMV-Tat plasmid (62) as a template. Next, PCR-generated myc-tagged *Vpu*, *Vpr*, *Vif*, and *Tat* cDNA cassettes were subcloned into the SpeI and XhoI sites of the lentiviral vector pTRIP-CMV-MCS-IRES-*Aequorea coerulescens* GFP (AcGFP) (63), whereas myc-tagged *Nef* cDNA was subcloned into the BamHI and SalI sites of this lentiviral vector. The resulting plasmid, pTRIP-AcGFP-Vpu, -Vpr, -Vif, -Nef, or -Tat, directed the independent expression of AcGFP and each viral protein.

**TABLE 1:** Oligonucleotide primers

Primer	Sequence^*a*^	Restriction enzyme
Vif forward	5′-CTAT**ACTAGT**CCGCCACCATGGAAAACAGATGGCAGGTGATG-3′	SpeI
Vif-myc reverse	5′-T**CTCGAG**TCA*CAGATCCTCTTCTGAGATGAGTTTTTGTTC*GTGTCCATTCATTGTATGGC-3′	XhoI
Vpr forward	5′-CTAT**ACTAGT**CCGCCACCATGGAACAAGCCCCAGAAGACC-3′	SpeI
Vpr-myc reverse	5′-T**CTCGAG**TCA*CAGATCCTCTTCTGAGATGAGTTTTTGTTC*GGATCTACTGGCTCCATTTC-3′	XhoI
Vpu forward	5′-CTAT**ACTAGT**CCGCCACCATGCAACCTATAATAGTAGC-3′	SpeI
Vpu-myc reverse	5′-T**CTCGAG**TCA*CAGATCCTCTTCTGAGATGAGTTTTTGTTC*CAGATCATCAATATCCC-3′	XhoI
Nef forward	5′-CTAT**GGATCC**GCCACCATGGGTGGCAAGTGGTCAAA-3′	BamHI
Nef-myc reverse	5′-T**GTCGAC**TCA*CAGATCCTCTTCTGAGATGAGTTTTTGTTC*GCAGTTCTTGAAGTACTCCG-3′	SalI
Tat forward	5′-CTAT**ACTAGT**CCGCCACCATGGAGCCAGTAGATCCTAG-3′	SpeI
Tat-myc reverse	5′-T**CTCGAG**TCA*CAGATCCTCTTCTGAGATGAGTTTTTGTTC*TTCCTTCGGGCCTGTCG-3′	XhoI

a Restriction endonuclease sites are in boldface type and underlined, and the myc tag is italicized.

### Vpu constructs.

The Vpu-encoding plasmids pCGCG-Vpu-AU1 (WT) and pCGCG-Vpu-AU1-S52,56D (64) were gifts of Daniel Sauter and Frank Kirchhoff (Institute of Molecular Virology, Ulm, Germany), and the proviral constructs pNL4.3, pNL4.3-VpuS52,56D, pNL4-3-VpuS52,56N, pNL4-3ADA.GFP.IRES.Nef, pNL4-3ADA.GFP.IRES.ΔNef, and pNL4-3ADA.GFP.IRES.NefΔVpu were previously described (28).

### Production of lentiviruses for expression of HIV-1 proteins.

HEK293T cells (2 × 10^6^) in 100-mm dishes were cotransfected with pTRIP-AcGFP plasmids for myc-tagged viral cDNAs (*Vpu*, *Vpr*, *Vif*, *Nef*, and *Tat*), pGag-Pol, and pHCMV-VSVG using PerFectin transfection reagent. Polybrene (4 μg/ml) and HEPES (20 mM) were added after 48 h to the lentivirus-containing culture supernatants, which were then passed through a 0.45-μm filter before aliquoting. Stocks were stored at −80°C or used immediately to transduce HeLa-CD4/CXCR4/CCR5 cells. Typically, lentiviral stocks were diluted 1:10 in DMEM containing 3% FBS, Polybrene (4 μg/ml), and HEPES (20 mM). Cells were then spinoculated by centrifugation at 1,200 rpm in an Eppendorf A-4-62 rotor for 1 h at 37°C, after which the plates were transferred to a 37°C incubator. After 6 h, the media were replaced with DMEM containing 10% FBS. Unless otherwise indicated, transduced cells were analyzed at 48 h posttransduction.

### Immunoblotting.

Transfected, transduced, or HIV-infected cells grown in 6-well plates were washed twice with cold PBS on ice and then lysed with radioimmunoprecipitation assay (RIPA) buffer (50 mM Tris-HCl [pH 7.4], 150 mM NaCl, 1% Triton X-100, 1% sodium deoxycholate, 0.1% SDS, 1 mM EDTA) containing a cocktail of protease inhibitors. Lysates were incubated on ice for 30 min and then centrifuged at 14,000 × *g* for 15 min at 4°C, after which protein concentrations in the supernatants were quantified using a Pierce bicinchoninic acid (BCA) protein assay kit (Thermo Fisher Scientific, Waltham, MA). Equivalent amounts of total protein (20 μg) were resolved by SDS-PAGE and then transferred to polyvinylidene difluoride membranes (Millipore Sigma) for immunoblotting.

Membranes were blocked with 3% skim milk powder in PBS containing 0.1% Tween 20 (PBS-T) and then incubated overnight at 4°C or for 3 h at room temperature with the appropriate primary antibodies diluted in 3% milk–PBS-T. After washing three times with PBS-T for 10 min each, fluorescent secondary antibodies (donkey anti-mouse IgG conjugated to Alexa Fluor 680 or goat anti-rabbit IgG conjugated to Alexa Fluor 680) diluted in PBS-T were used to detect the primary antibodies. After a 1-h incubation with the secondary antibodies, membranes were washed three times with PBS-T for 10 min each. Detection and quantification of the protein signals in the immunoblots were performed using a Licor (Lincoln, NE) Odyssey infrared imaging system according to the manufacturer’s protocol (LI-COR Biosciences; https://www.licor.com/documents/fxc6evxvxbub4srkqy6i9yg46l7i0xz5). Relative levels of PEX2, PEX7, PEX11B, PEX13, and catalase (normalized to actin) were determined using Odyssey infrared imaging system version 1.2 software.

### Confocal microscopy.

HeLa-CD4/CXCR4/CCR5 cells grown on coverslips were processed for confocal microscopy at 48 h or 72 h posttransfection or postinfection. Cells were washed in PBS containing 0.5 mM Ca^2+^ and 1 mM Mg^2+^ and then fixed with 3% paraformaldehyde (for confocal imaging) or 1.5% electron-microscopy-grade paraformaldehyde (for superresolution imaging) for 30 min at room temperature. Samples were then quenched with 50 mM NH_4_Cl in PBS for 5 min at room temperature, washed three times with PBS, and then permeabilized with 0.2% Triton X-100 for 5 min. Incubations with primary antibodies diluted (1:500 to 1:1,000) in blocking buffer (3% bovine serum albumin [BSA] in PBS) were performed at room temperature for 2 h, followed by three washes in PBS containing 0.1% BSA. Samples were then incubated with secondary antibodies in blocking buffer for 1 h at room temperature, followed by three washes in PBS containing 0.1% BSA. Secondary antibodies were donkey anti-mouse/anti-rabbit IgG conjugated to Alexa Fluor 488 and donkey anti-mouse IgG conjugated to Alexa Fluor 546.

Coverslips were mounted onto microscope slides using ProLong gold antifade reagent with DAPI, and samples were examined using an Olympus 1x81 spinning-disc confocal microscope equipped with a 60×/1.42 oil PlanApo N objective. Confocal images were acquired and processed using Volocity 6.2.1 software.

### Quantification of peroxisomes.

The z-stack images acquired using a confocal microscope were exported from Volocity 6.2.1 as an OEM.tiff file. The exported images were then processed using Imaris 7.2.3 software (Bitplane). Peroxisomes within polygonal areas that excluded the nucleus were quantified (quality and voxel). Within the selected regions, the absolute intensity/region volumes of the peroxisomes were determined and then entered into a Microsoft Excel spreadsheet. Only those SKL/PMP70-positive structures with volumes of between 0.001 and 0.05 μm^3^ were included for measurement. The data were then analyzed using Student’s *t* test.

### qPCR analysis of miRNA expression.

Total RNAs, including small RNA from transfected, lentivirus-transduced, or HIV-infected HeLa-CD4/CXCR4/CCR5 cells, primary MDMs, and lymphocytes, were purified using the miRNeasy minikit (Qiagen) according to the manufacturer’s instructions. Mature miRNAs and certain small nucleolar RNAs (snoRNAs) and small nuclear RNAs (snRNAs) were selectively reverse transcribed into cDNA using miScript HiSpec buffer according to the instructions of the miScript II RT kit (Qiagen). Briefly, mature miRNAs were polyadenylated by poly(A) polymerase and reverse transcribed into cDNA using oligo(dT) primers. Polyadenylation and reverse transcription were performed in parallel in the same reaction. The oligo(dT) primers included a 3′ degenerate anchor and a universal tag sequence on the 5′ end, allowing the amplification of mature miRNA in the real-time PCR step.

The resulting cDNAs served as the templates for real-time PCR analysis using miRNA-specific forward primers (IDT) and the miScript SYBR green PCR kit (Qiagen), which contains the miScript universal primer (reverse primer) and QuantiTect SYBR green PCR master mix. The amplification cycles consisted of an initial activation step at 95°C for 15 min, followed by 40 cycles of 15 s at 94°C, 30 s at 55°C, and 30 s at 70°C. Fluorescence data were collected during the 70°C extension step. The miRNA targets and primers that were used in this study were previously described (11). As an internal control, the levels of a small nuclear RNA, RNU6B (a miScript PCR control provided in the miScript PCR starter kit [Qiagen]), were determined. Relative miRNA expression was normalized to RNU6B levels using the comparative threshold cycle (ΔΔ*C_T_*) method. All miRNA expression studies were conducted using an Mx3005P thermocycler (Stratagene, La Jolla, CA).

### Measurement of nonesterified fatty acids.

Levels of NEFAs in Vpu-expressing and HIV-1-infected HeLa-CD4/CXCR4/CCR5 cells were measured using a modified copper-triethanolamine assay as described previously (65). Parent HeLa-CD4/CXCR4/CCR5 and PEX19 knockout HeLa-CD4/CXCR4/CCR5 cells were transfected with the Vpu-encoding plasmid pCGCG-Vpu-AU1 or an empty vector (pCGCG) using Lipofectamine 2000 (Invitrogen). At 48 h posttransfection, cells (∼1 × 10^7^) were collected and homogenized in 200 μl of chloroform containing 1% Triton X-100 and then subjected to centrifugation at 13,000 × *g* for 10 min. The supernatants were removed and then evaporated at 60°C. Lipids were taken up in 200 μl of PBS, after which 25-μl aliquots were transferred to glass vials containing 500 μl of chloroform-heptane (4:3 ratio). Vials were shaken for 2 min and then centrifuged for 5 min at 2,000 × *g*. Three hundred microliters of the organic phases was transferred to new glass vials containing 250 μl of copper-triethanolamine, which were shaken for 2 min before centrifugation for 5 min at 2,000 × *g*. Copper-triethanolamine was made by combining 10 ml of 0.5 M Cu(NO_3_)_2_, 10 ml of 1.0 M triethanolamine, and 3.5 ml of 1 M NaOH and then diluted with water to 100 ml, after which 33 g of NaCl was added and the pH was adjusted to 8.1. Organic-phase aliquots (150 μl) were again collected and evaporated at 60°C in glass vials, after which the remaining lipids were solubilized with ethanol (150 μl), with shaking for 15 min at 37°C. Copper was detected in the samples by complexation with a mixture of dicarbazone-dicarbazide (100 μl), and the color intensity was measured in a 96-well plate at 550 nm in a microplate reader (BioTek). A NEFA standard solution (Fuji Film Wako) was used for quantitation, and the amounts of NEFAs relative to the protein content in the sample (measured using a Bio-Rad protein assay kit) were determined.

Infection of parent and PEX19 knockout HeLa-CD4/CXCR4/CCR5 cells with HIV-1 (NL4-3) was performed under biosafety CL3 conditions at an MOI of 3 for 72 h, after which the concentrations of NEFAs were determined.

### Generation of PEX19 knockout cells by CRISPR.

PEX19 knockout cells were generated by using the Alt-R CRISPR-Cas9 system from IDT (Coralville, IA).

**(i) Transfection of HeLa-CD4/CXCR4/CCR5 cells with a Cas9-crRNA-tracrRNA ribonucleoprotein complex.** Briefly, Alt-R CRISPR crRNA specific for PEX19 (Hs.Cas9.PEX19.1.AA [sequence, AltR1-rGrG rArArC rUrArU rUrCrG rArCrA rGrUrG rArArC rGrUrU rUrUrArGrArG rCrUrA rUrGrC rU-AltR2]) and tracrRNA (fluorescence-labeled Atto 550) were resuspended separately in nuclease-free duplex buffer to a final concentration of 100 μM. CRISPR RNA (crRNA) and *trans*-activating crRNA (tracrRNA) were diluted to 1 μM and then annealed by heating to 95°C for 5 min, followed by cooling to room temperature. To produce the ribonucleoprotein (RNP) for each well in 96-well plates, 4.5 μl of 1 μM crRNA-tracrRNA mix was combined with 4.5 μl Cas9 enzyme (1 μM) and 16 μl Opti-MEM (Thermo Fisher). The mixtures were incubated at room temperature for 5 min to allow the assembly of the RNP complexes. Alt-R CRISPR-Cas9 controls (negative and positive [hypoxanthine phosphoribosyltransferase {HPRT}]) were also included in the experiments. Next, the RNP complexes were reversed transfected as follows. For each well in the 96-well plate, 25 μl of RNP, 2 μl of Lipofectamine RNAiMAX transfection reagent (Thermo Fisher), and 23 μl of Opti-MEM (Thermo Fisher) were combined and incubated at room temperature for 20 min to form transfection complexes. When the incubation was complete, 50 μl of transfection complexes was added to the wells of a 96-well tissue culture plate. Next, 100 μl of suspended HeLa-CD4/CXCR4/CCR5 cells (40,000 cells/well) was added to each well of the transfection mixture (the final concentration of RNP was 30 nM). The plate containing the transfection complexes and cells was incubated in a cell culture incubator (37°C with 5% CO_2_) for 48 h.

**(ii) Mutation detection by T7EI mismatch endonuclease.** Genomic DNA from CRISPR-Cas9-edited cells was isolated by the addition of 50 μl of QuickExtract DNA extraction solution (Epicentre) and then transferred to PCR tubes. The DNA was vortexed and then heated at 65°C for 10 min, followed by 98°C for 5 min, in a thermal cycler. Following heating, the genomic DNA was diluted with 100 μl of nuclease-free water. CRISPR target sites (PEX19 and controls) were then amplified with a platinum *Taq* DNA polymerase PCR kit (Thermo Fisher). Heteroduplexes were formed by denaturing at 95°C and cooling slowly to room temperature. Detection of CRISPR editing was done by visualization of T7EI cleavage products with an Alt-R genome editing detection kit (IDT). Primers used were PEX19 CRISPR FWD Set 4 (sequence, CAC GGA CTC TGC ATC AGT TTA) and PEX19 CRISPR REV Set 4 (sequence, CCA TGT CTC TTG TCT CTG AAG G). Single-cell sorting was also performed 48 h after transfection, and PEX19 knockout in the cells was further confirmed by Western blotting and confocal imaging.
