# Supplementary figures and images for: The HIV-1 Accessory Protein Vpu Downregulates Peroxisome Biogenesis
Source: mBio. 2020 Mar 3;11(2):e03395-19. doi: 10.1128/mBio.03395-19 (PMC7064786; doi:10.1128/mBio.03395-19)

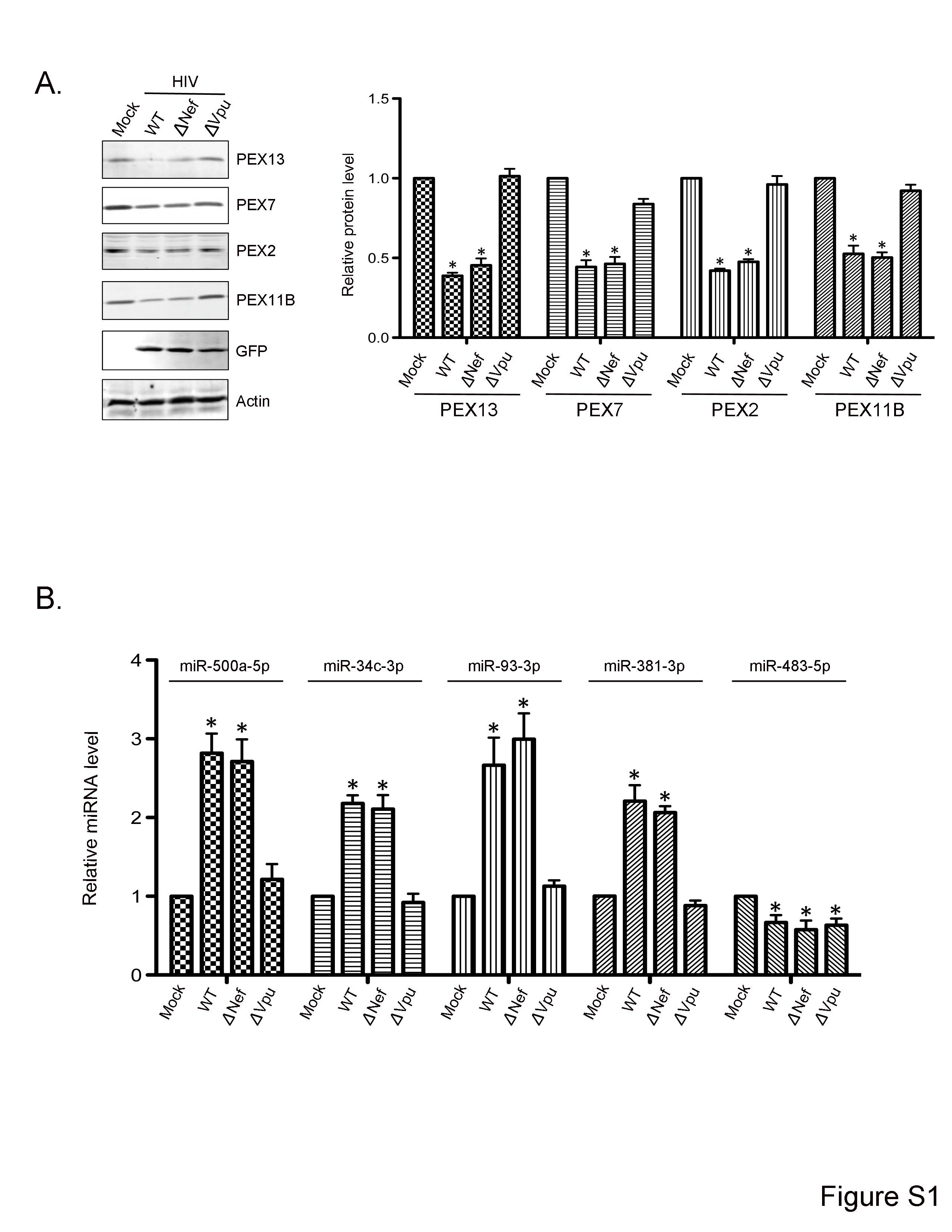

Supplement: FIG S1 [file mBio.03395-19-sf001.tif]

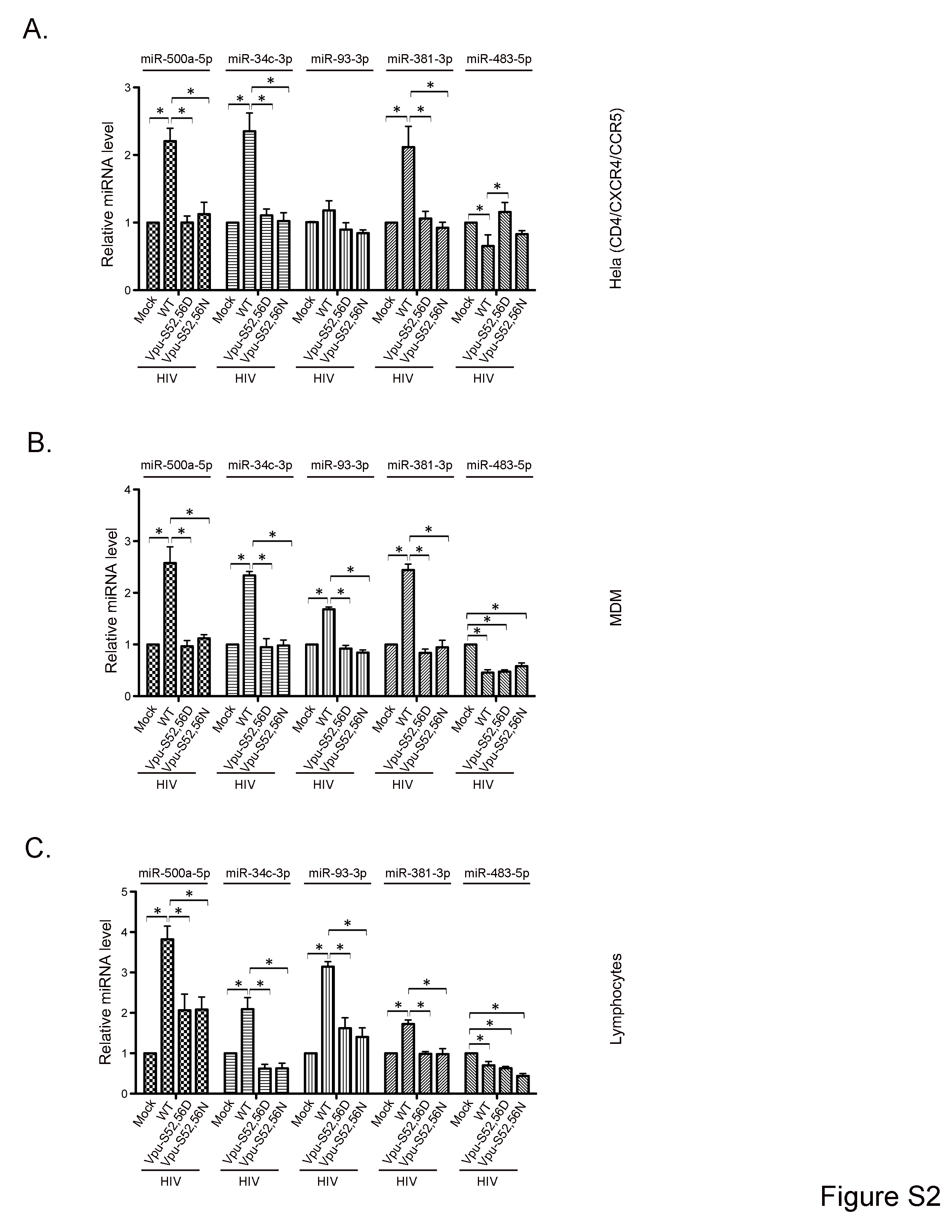

Supplement: FIG S2 [file mBio.03395-19-sf002.tif]

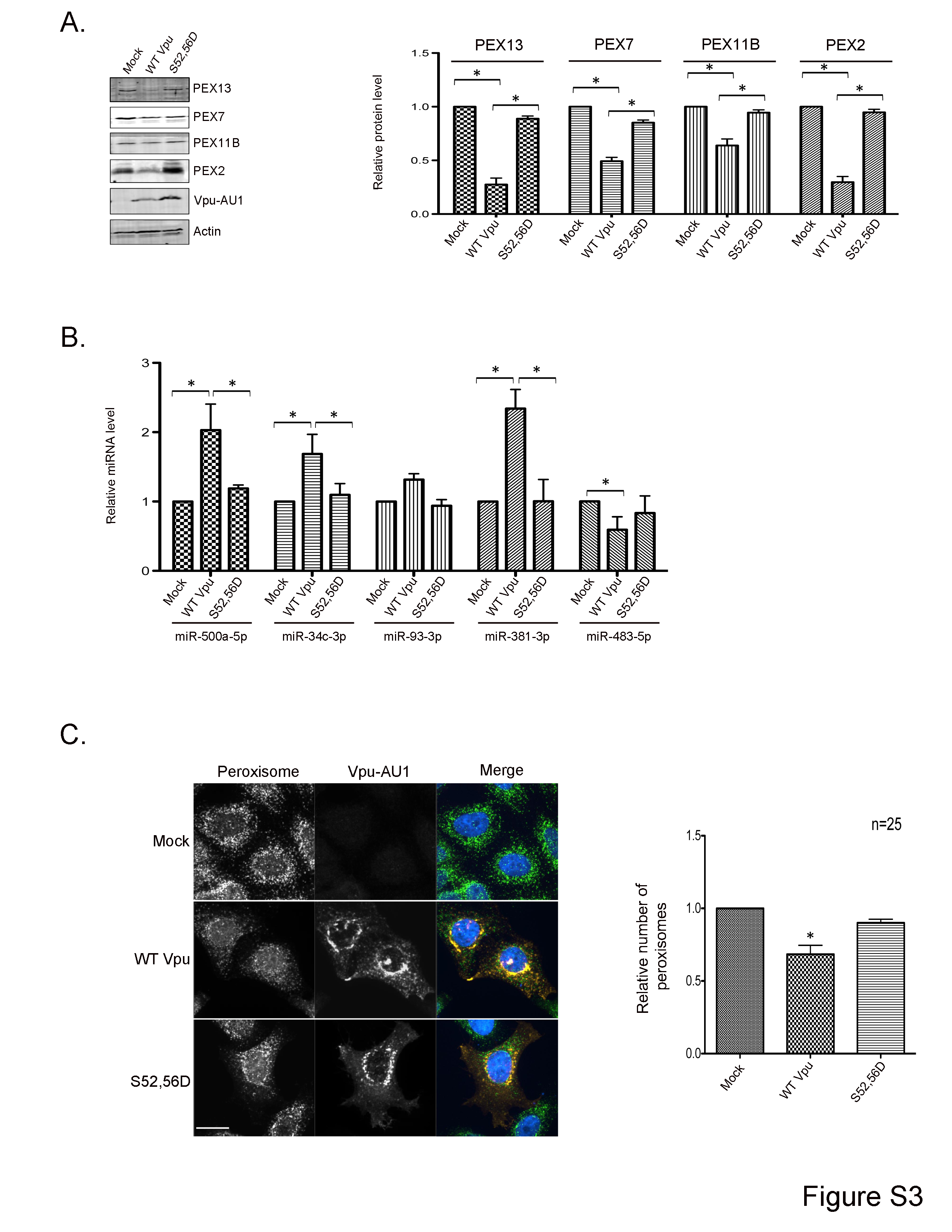

Supplement: FIG S3 [file mBio.03395-19-sf003.tif]
